# Powder Bed Fusion 3D Printing in Precision Manufacturing for Biomedical Applications: A Comprehensive Review

**DOI:** 10.3390/ma17030769

**Published:** 2024-02-05

**Authors:** Rajan John Nekin Joshua, Sakthivel Aravind Raj, Mohamed Thariq Hameed Sultan, Andrzej Łukaszewicz, Jerzy Józwik, Zbigniew Oksiuta, Krzysztof Dziedzic, Arkadiusz Tofil, Farah Syazwani Shahar

**Affiliations:** 1 Department of Manufacturing Engineering, School of Mechanical Engineering, Vellore Institute of Technology, Vellore 632014, Tamil Nadu, India; nekinjoshua.r@gmail.com; 2 Department of Aerospace Engineering, Faculty of Engineering, Universiti Putra Malaysia, Serdang 43400, Selangor, Malaysia; farahsyazwani@upm.edu.my; 3 Laboratory of Biocomposite Technology, Institute of Tropical Forestry and Forest Products (INTROP), Universiti Putra Malaysia, Serdang 43400, Selangor, Malaysia; 4 Aerospace Malaysia Innovation Centre (944751-A), Prime Minister’s Department, MIGHT Partnership Hub, Jalan Impact, Cyberjaya 63000, Selangor, Malaysia; 5 Institute of Mechanical Engineering, Faculty of Mechanical Engineering, Bialystok University of Technology, Wiejska 45C, 15-351 Bialystok, Poland; a.lukaszewicz@pb.edu.pl; 6 Department of Production Engineering, Faculty of Mechanical Engineering, Lublin University of Technology, Nadbystrzycka 36, 20-618 Lublin, Poland; j.jozwik@pollub.pl; 7 Institute of Technical Sciences and Aviation, University College of Applied Sciences in Chełm, Pocztowa 54, 22-100 Chełm, Poland; atofil@panschelm.edu.pl; 8 Institute of Biomedical Engineering, Faculty of Mechanical Engineering, Bialystok University of Technology, Wiejska 45C, 15-351 Bialystok, Poland; z.oksiuta@pb.edu.pl; 9 Institute of Computer Science, Electrical Engineering and Computer Science Faculty, Lublin University of Technology, Nadbystrzycka 36, 20-618 Lublin, Poland; k.dziedzic@pollub.pl

**Keywords:** powder bed fusion, biomedical applications, additive manufacturing, implants, precision manufacturing

## Abstract

Precision manufacturing requirements are the key to ensuring the quality and reliability of biomedical implants. The powder bed fusion (PBF) technique offers a promising solution, enabling the creation of complex, patient-specific implants with a high degree of precision. This technology is revolutionizing the biomedical industry, paving the way for a new era of personalized medicine. This review explores and details powder bed fusion 3D printing and its application in the biomedical field. It begins with an introduction to the powder bed fusion 3D-printing technology and its various classifications. Later, it analyzes the numerous fields in which powder bed fusion 3D printing has been successfully deployed where precision components are required, including the fabrication of personalized implants and scaffolds for tissue engineering. This review also discusses the potential advantages and limitations for using the powder bed fusion 3D-printing technology in terms of precision, customization, and cost effectiveness. In addition, it highlights the current challenges and prospects of the powder bed fusion 3D-printing technology. This work offers valuable insights for researchers engaged in the field, aiming to contribute to the advancement of the powder bed fusion 3D-printing technology in the context of precision manufacturing for biomedical applications.

## 1. Introduction

### 1.1. Additive Manufacturing

**Figure 1:**
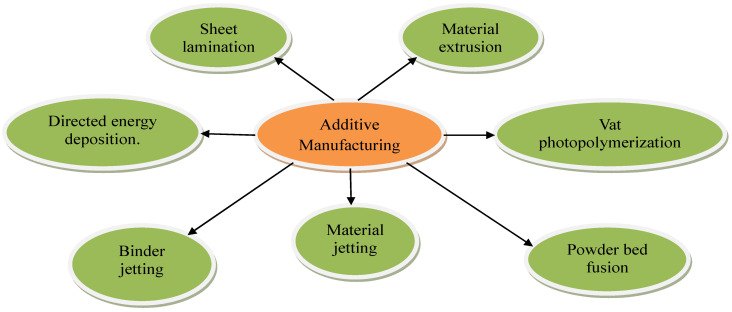
Types of additive-manufacturing technologies.

Additive-manufacturing (AM) technology, often known as 3D printing [1], is a manufacturing process that stands in contrast to subtractive-manufacturing methods. AM is the process for adding materials layer by layer instead of material removal. AM offers a wide range of opportunities for industries to manufacture complex parts without joints. Based on ISO/ASTM 52900:2021 [2], AM is further classified into seven major types [1,3], as shown in Figure 1. They are material extrusion, vat photopolymerization, powder bed fusion, material jetting, binder jetting, sheet lamination, and directed energy deposition [4].

Fused-deposition modeling, also referred to as material extrusion, involves the continuous extrusion of a molten thermoplastic filament to produce the desired object [5]. Fused-deposition modeling (FDM) is commonly used for prototyping, specific manufacturing, and small-scale production. Additionally, it is used in biomedical applications, such as drug delivery systems, dental implants, orthotics, prostheses, FDM-printed bone models, and bioprinting scaffolds [6,7,8,9,10].

Vat photopolymerization uses a liquid photopolymeric resin to generate the final product. The liquid photopolymeric resin is cured layer by layer using ultraviolet light produced by a laser system [11]. The applications of vat photopolymerization include the manufacturing of high-detail prototypes and jewelry and the small-scale manufacturing of complex parts. Additionally, drug delivery systems, dental implants, crowns, bone models, microfluidic devices, and scaffolds for tissue engineering are among the biomedical applications [12,13,14,15,16].

Material jetting is similar to inkjet printing. It uses photopolymeric materials instead of ink on a build platform and then cures them with ultraviolet light [17]. The applications of material jetting include the production of high-resolution prototypes and intricate multi-material products. In the biomedical field, it finds use in drug delivery systems that utilize multiple materials, bio-printed tissues and scaffolds, surgical models and guidance, and more [18,19,20].

Binder jetting is a unique AM process that is used to manufacture final items with a powdered material and a binding agent [21]. The applications of binder jetting include the production of sand-casting molds, metal components, and full-color prototypes. Additionally, it has biomedical applications, such as the creation of bone scaffolds, surgical models, drug delivery devices, and dental-casting models [18,22,23,24].

Sheet lamination involves the sequential stacking of materials, followed by the application of heat and an adhesive to securely fuse the layers and create finished items [25]. The applications of sheet lamination AM include prototyping, manufacturing large-format products, and working with composite materials and biomedical applications, such as surgical models and phantoms [26].

Directed energy deposition utilizes focused heat energies, such as those from lasers or electron beams, to melt and fuse materials as they are being deposited. The applications of directed energy deposition include the repair and modification of existing parts, as well as the AM of large structures through layer-by-layer deposition and biomedical applications, such as tissue engineering and dosage forms [27,28]. Many different biomaterials, like polymers, hydrogels, ceramics, alloys, and metals, have been made with AM [29].

PBF has gained popularity in the biomedical field for a number of reasons, including material versatility: PBF technology demonstrates material diversity by employing polymers, ceramics, and metals. The diverse range of materials enables the customization of medical devices and implants to meet the specific requirements of individual customers [30]. Exceptional accuracy: PBF technology has the ability to manufacture components with remarkable precision, including those with delicate features. Precision and customization are vital in the biomedical industry [31,32]. Strength and long life: PBF-printed parts exhibit high strength and durability, rendering them well suited for load-bearing applications, such as orthopedic implants [33]. Ability to work with multiple materials: The PBF method has the capability to produce parts possessing qualities derived from multiple materials. It is advantageous for fabricating biomedical devices possessing qualities peculiar to certain regions [34,35].

Comparatively, other techniques have certain limitations. The material extrusion process results in poor surface quality and is limited to thermoplastic materials [36]. The sheet lamination technique can be used with a limited number of materials and results in lower surface quality [37]. Although residual powders can be recycled, their mechanical properties may not be equivalent to those processed using PBF [38]. Material-jetting, vat photopolymerization, and direct-energy-deposition technologies lack the equivalent levels of material diversity, efficiency, and intricacy compared with those of PBF [39].

**Figure 2:**
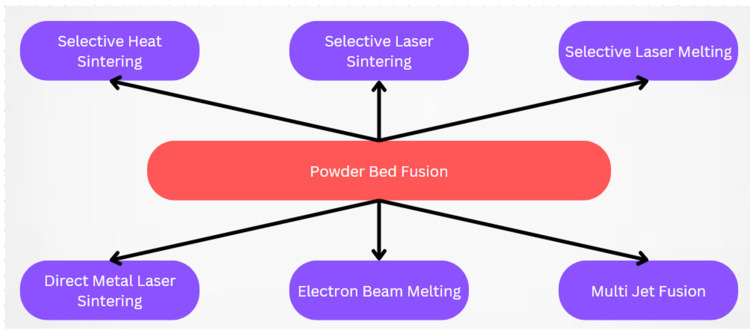
Different PBF Technologies.

This review focuses on the PBF method, which is closely related to precision component manufacturing. PBF uses a heat source to fuse powdered materials together and possesses the capability to manufacture intricate metallic components with a notable degree of dimensional precision [1]. The most significant PBF-based processes include SHS, SLS, SLM, DMLS, EBM, and MJF, as shown in Figure 2.

The benefits of PBF technologies include reduced material waste, part customization, low-volume production, and the production of complex parts.

### 1.2. The Significance of Precision Manufacturing in the Biomedical Sector

Precision manufacturing is considered in the biomedical field owing to the requirements of durability and a high level of precision. Several researchers are currently engaged in biomedical research endeavors aimed at the precise additive manufacturing of functional components within the human body [40,41]. The implementation of this approach guarantees the manufacturing of accurate medical equipment, diagnostics, and individualized treatments, resulting in enhanced patient outcomes, greater diagnostic capabilities, and compliance with rigorous regulatory requirements.

## 2. PBF 3D Printing

### 2.1. Overview

**Figure 3:**
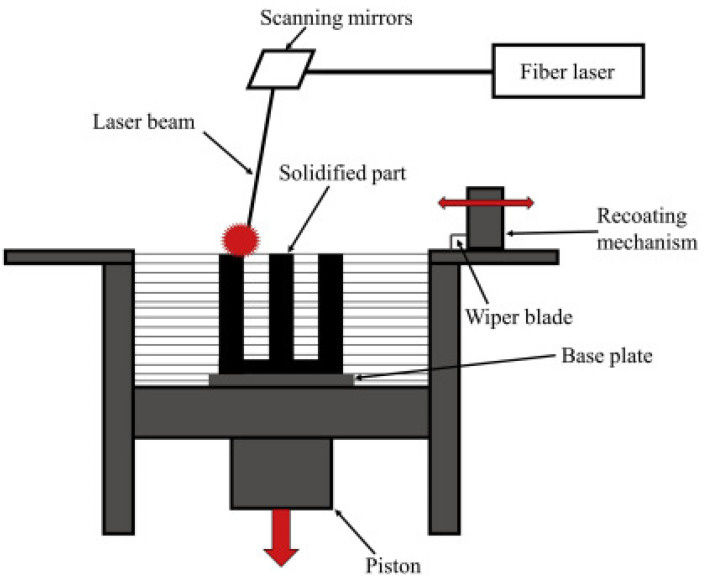
SLM-based PBF 3D printing working principle, adapted and modified from [43].

Powdered materials are used in PBF, an additive manufacturing technique, to build an object layer by layer. The powdered materials may be polymers or metals. The first step in the PBF process is slicing. A computer-modeled 3D file is sliced with the help of slicing software. During the slicing process, the 3D model is sliced into several individual layers. In the second step, a thin layer of material is spread over a build platform. The powder particles are spherical in shape, and the particle sizes used for SLM, SLS, and EBM are, respectively, 15–40 µm, 20–80 µm, and 40–100 µm [42]. In the third step, a laser or electron beam is used to fuse the powder materials, and the laser or electron beam follows the path of the provided 3D model. In SLS, a laser is used to heat materials below their melting points, allowing the materials to melt completely and fuse together to form the dense parts of metals. In the fourth step, the build platform is lowered by a thickness of one layer after completing the first layer of the sliced 3D model. In the process, steps two to four are repeated until the completion of the entire object. Figure 3 shows the schematic diagram of the PBF technology. The fifth step is post-processing. In this post-processing step, the unused powders are collected for reuse. The final product is then removed for a further heat treatment process if required.

#### 2.1.1. Selective Heat Sintering

SHS is a form of PBF additive-manufacturing technology that is used to fuse powdered thermoplastic materials layer by layer to generate a finished product using a thermal printhead. It is comparable to SLS, but SHS employs a more portable and reasonably priced thermal printhead in place of a powerful laser [44,45]. The typical range for layer thickness is 50–200 µm [44,45].

A 3D model is generated utilizing CAD software and subsequently divided into tiny layers through the utilization of slicing tools. The powdered thermoplastic is applied to the build platform using a roller. The thermal printhead directs heat to specific areas of the powder bed according to the shape of the layer being printed. The heat facilitates the fusion of the powder particles that are below the melting point, thereby enabling the formation of the intended shape. Once the single layer is completed, for the next layer, the powder is spread over the build platform with the help of a roller. The process is repeated until the completion of the final product [46].

The cost effectiveness of SHS is attributed to its utilization of a thermal printhead instead of a laser, resulting in lower prices compared with those for using SLS printers [46]. SHS printers are more compact and desktop friendly than SLS printers [45]. SHS exhibits material adaptability, as it may be effectively utilized with a diverse array of thermoplastic materials, such as nylon, polyamide, and polystyrene [47]. The drawbacks of SHS include reduced printing speed, unsuitability for high-temperature applications, and the restricted availability of SHS 3D printers [48].

#### 2.1.2. Selective Laser Sintering

**Figure 4:**
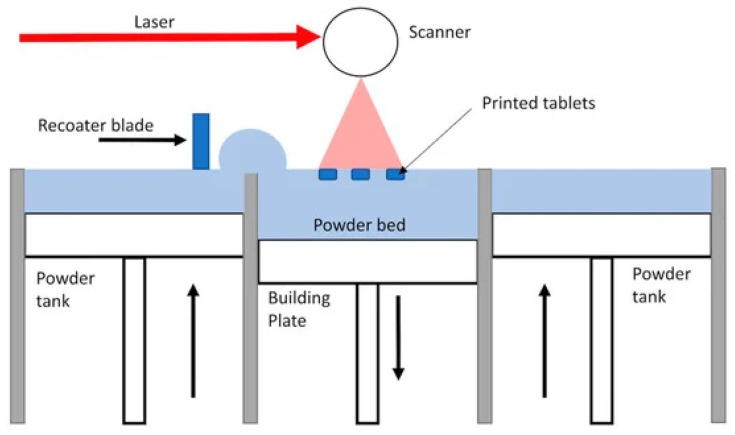
Schematic diagram of SLS-based PBF 3D-printing process, adapted from [58].

The SLS additive-manufacturing process uses metallic powders, like titanium [49] and stainless steel [50]; ceramics, like alumina and zirconia [51]; and powdered thermoplastic materials, like nylon [52], thermoplastic polyurethane [53], and polyamide [54], to create final components using a powerful laser source. The process of SLS involves the slow fusion of small powder particles layer by layer to create the required shape [55,56,57]. The schematic diagram of the SLS 3D-printing process is shown in Figure 4.

The initial step is designing the 3D model using CAD software, followed by slicing the model into several layers using slicing software. This slicing process establishes a trajectory for the laser to track. Next, a fine coating of powder is evenly distributed throughout the build platform using either a roller or a recoater blade. The laser is precisely directed along the predetermined trajectory on the powder, resulting in the fusion of powder particles and the completion of the first layer [59]. Following the completion of the first layer, the build platform is lowered, and the powder recoater distributes the powder for printing the subsequent layer. The powder particles and fused powders are once more the focus of the laser. The procedure is repeated until the finished product is completed [49]. For example, Figure 4 shows the printing of tablet shapes. [58]. In certain instances, the presence of a support structure is necessary. The thickness of a layer normally falls in the range 20–150 µm [55]. The primary benefit of SLS is the ability to create complex structures with minimum support requirements, which streamlines the postprocessing phase and minimizes material waste. The SLS technology is very proficient in manufacturing intricate shapes and making long-lasting and practical components. Nevertheless, the utilization of high-power lasers in SLS might result in increased costs [60].

#### 2.1.3. Selective Laser Melting

**Figure 5:**
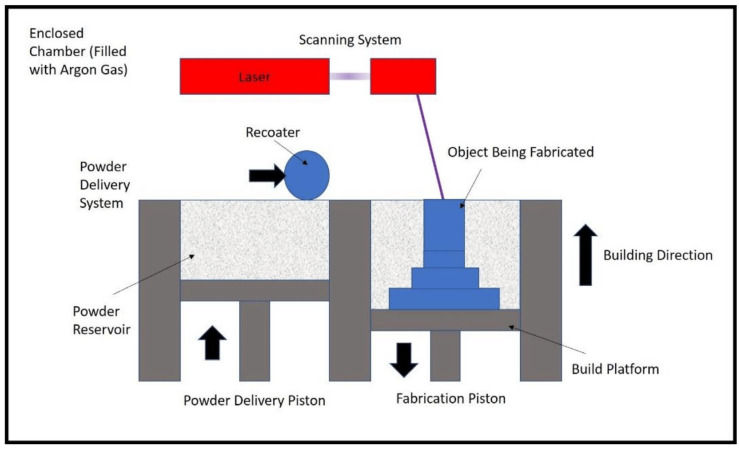
Schematic diagram of SLM-based PBF 3D-printing process, adapted from [67].

SLM is a manufacturing process that employs a high-power laser source to achieve the complete melting of metallic powders, resulting in the production of dense and robust components possessing exceptional mechanical characteristics. Aluminum, stainless steel, and titanium are the metallic powders that are the most used in SLM [61,62,63,64,65]. SLM is widely used in industries that require high-performance metal components, such as aerospace and biomedical. Typically, the thickness of a layer ranges from 50 to 75 µm [66]. SLM produces a surface quality that is superior to those produced using SHS and SLS. The schematic diagram of the SLM-based 3D-printing process is shown in Figure 5.

Initially, a 3D model is created using CAD software and then divided into several layers using slicing tools. As shown in Figure 5, a thin layer of metallic powder is spread over the build platform with the help of a recoater. A high-power laser is precisely directed toward the powder bed [59]. During that period, the metallic powders undergo the process of melting and fusion, resulting in the bonding of the desired particles [68]. This process solidifies the first layer. After the initial layer is finished, the fabrication piston is lowered, the powder delivery piston goes up, and the recoater redistributes the powder. Once more, the laser is directed at the powder bed. The procedure is repeated until the attainment of the final product. Argon gas is used in the enclosed chamber to prevent the parts from oxidizing [69]. The benefits of SLM include the ability to achieve design flexibility, produce robust and durable parts, work with a wide range of materials, and minimize waste [70,71]. The limitations of SLM are its excessive cost, restricted building capacity, and necessity for support structures.

#### 2.1.4. Direct Metal Laser Sintering

**Figure 6:**
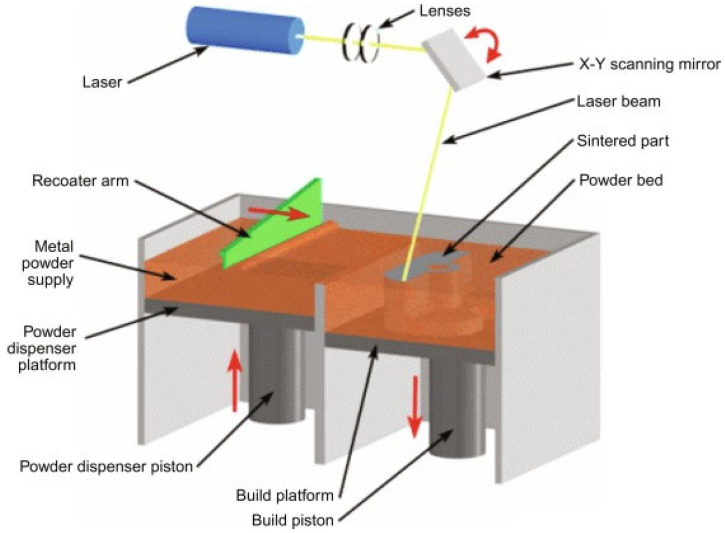
Schematic diagram of DMLS-based PBF 3D-printing process, adapted from [77].

DMLS is an additive-manufacturing technology that emphasizes the sintering aspect instead of full melting. A laser is used in DMLS to selectively fuse metallic particles. The thickness of a layer ranges between 20 and 40 µm [72]. The commonly used materials are stainless steel, nickel alloys, copper, titanium, and aluminum [73,74,75,76]. The printing process is quicker than that in SLM. It is also used in industries that require high-performance metal components, such as aerospace and biomedical. Owing to the use of thinner layers, DMLS is able to achieve a greater resolution than SLS [77]. The surface quality produced using DMLS is significantly higher than that produced using SLS. The schematic diagram of DMLS 3D printing is shown in Figure 6.

As shown in Figure 6, the laser is focused on the X-Y scanning mirror, which helps to control the laser beam’s direction. The laser selectively sinters the metallic powders available on the build platform according to the sliced CAD model. In preparation for the subsequent layer printing, the build platform is lowered using the build piston, the powder dispenser piston is lifted, and the metallic powder is delivered using the recoater’s arm. Once more, the laser is directed toward the build platform, and the process is repeated until the last layer is finished [78].

DMLS has several benefits, including exceptional accuracy and precision, the ability to create intricate shapes, the production of robust yet lightweight components, and a reduction in waste [79,80]. Nevertheless, the drawbacks of DMLS encompass its high expenses, lengthy production duration, and restricted range of available materials [81].

#### 2.1.5. Electron Beam Melting

**Figure 7:**
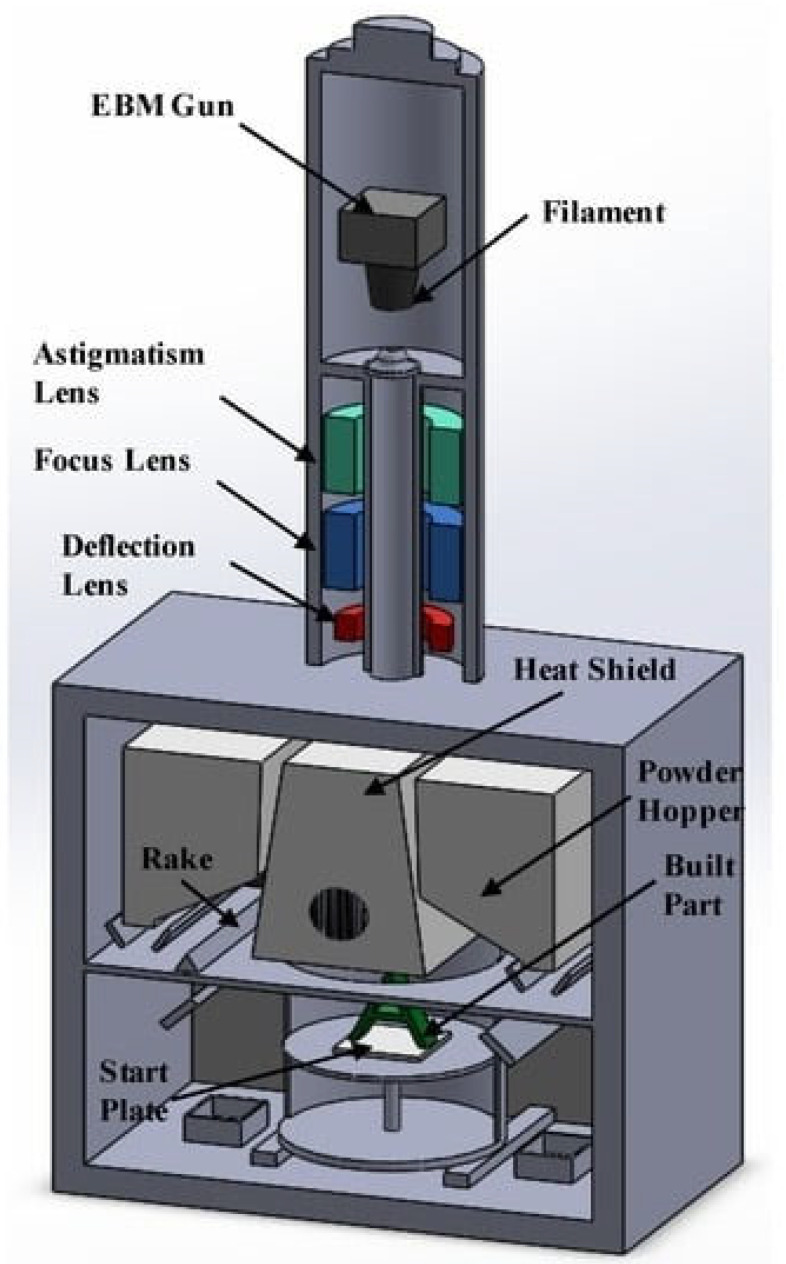
Schematic diagram of EBM-based PBF 3D-printing process, adapted from [87].

EBM is a manufacturing technique that employs an electron beam as opposed to a laser for the purpose of melting and fusing metallic powders [82]. EBM is conducted under vacuum conditions [83] to mitigate the effects of oxidation, hence rendering it a feasible method for fabricating aerospace-grade components with enhanced strength properties. The thickness of a layer ranges between 50 and 150 µm [83]. The commonly used metallic powders are nickel alloys, aluminum, stainless steel, and titanium [84,85,86]. The schematic diagram of the EBM 3D-printing process is shown in Figure 7.

CAD software is used for designing the model of the product, and slicing software is used to slice the model into many layers. As shown in Figure 7, the powder hopper transports metallic powder from its storage. The metallic powder is distributed over the start plate, which is normally composed of steel. Next, an electron beam gun is used to melt the metallic powder in a predetermined path [82]. The electron beam gun includes a filament, an anode, and a sequence of lenses that guide the electron beam. Following the completion of the initial layer printing, the start plate is then lowered, and a fresh layer of powder is evenly distributed over it. Once more, the electron beam gun concentrates its energy on the metallic powder, causing the powder to melt. The procedure is repeated until the completion of the final product [88].

#### 2.1.6. Multi-Jet Fusion

MJF is an innovative additive-manufacturing technology pioneered by HP that elevates the capabilities of 3D printing [89,90]. It is perfect for rapidly and economically generating high-quality prototypes and finished items. Commonly used materials include powdered nylon, thermoplastic polyurethane, polyamide, polycarbonate, and polypropylene. A 3D model is designed with the help of CAD software and sliced into a number of layers with the help of slicing software. The MJF printer uniformly distributes a fine layer of nylon powder (for example) on the build platform. A thermal inkjet array is used to selectively apply two agents to the powder bed: a fusing agent and a detailing agent. The fusing agent serves to adhere the powder particles together in the regions that align with the intended shape of the component [91,92]. The detailing agent functions as a protective barrier surrounding the fused regions, effectively avoiding undesired powder fusion and guaranteeing the preservation of precise edges and intricate details [93]. A heating element is used to scan the print bed and fuse the places where the fusing agent is applied [91,92]. After the layer is finished, the build platform lowers significantly. A layer of a new powder is evenly distributed on the surface, and the sequence for adding materials, heating, and lowering is repeated for each consecutive layer until the complete object is built.

The benefits of MJF 3D-printing technology encompass practical prototypes, the ability to print objects in color, good surface finishes, fine features, and the capability to recycle unused powder [94]. Constraints encompass the need for postprocessing and the restricted range of material choices [95].

### 2.2. Materials Used in PBF

PBF technology uses a wide range of materials, including metals, metal alloys, polymers, ceramics, and composites [96].

#### Materials Used in Biomedical Implants

The most used materials in biomedical implants are titanium and its alloys [97,98,99,100,101,102,103,104,105,106], Co-Cr-based alloys [107,108,109], NiTi alloy [110,111,112,113], stainless steel [114,115], magnesium and its alloys [116], and AlSi10Mg [117]. The advantages of the commonly used biomedical materials are listed in Table 1.

Materials are chosen based on the biomedical application and the specific manufacturing process. For long-term orthopedic implants, titanium can be used, whereas NiTi is used in stents. Additionally, temporary implants can be made of magnesium alloys.

## 3. Application of PBF 3D Printing in the Biomedical Field

### 3.1. Biomedical Implants

In biomedical implants, the use of PBF technology is more widespread. With the help of PBF technology, sophisticated body parts and implants made specifically for a patient can be produced.

**Figure 8:**
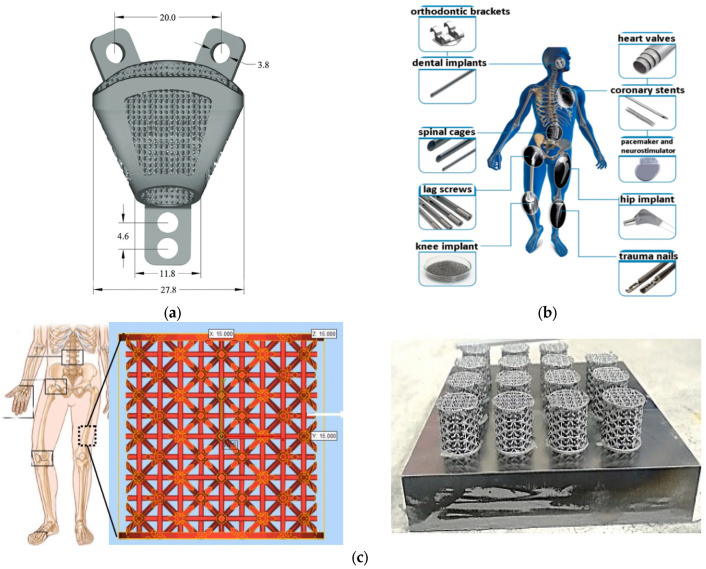

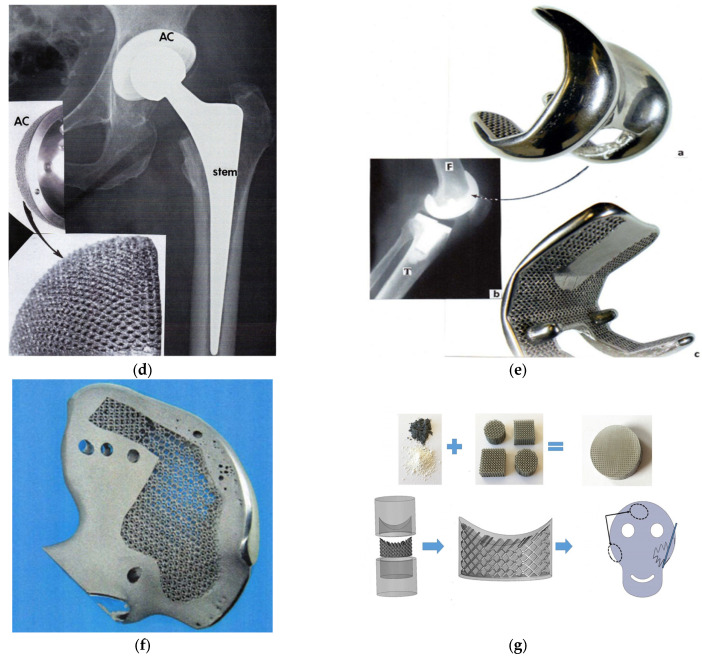
PBF-based 3D-printed biomedical implants (**a**) SLM 3D-printed spinal implant (dimensions are in mm) [139]; (**b**) titanium-based biomedical implants [140]; (**c**) SLM 3D-printed cellular implant plug design for osteoarthritis patients [104]; (**d**) X-ray image of total hip replacement, EBM 3D-printed acetabular cup (AC) made of Ti-6Al-4V [141]; (**e**) Illustration of a femoral appliance fabricated using Co-Cr-Mo EBM, for total knee replacement (implant). Parts a and c in the picture depict the external (contact surface) and the internal reticulated mesh (femoral end), respectively. Part b in the picture presents a typical X-ray image of a total knee replacement. The letters F and T are used to represent the femur and tibia, respectively [142]; (**f**) EBM-fabricated porous pelvic girdle, right iliac bone made of Ti-6Al-4V [142]; (**g**) SLM–SPS-fabricated cranio-maxillofacial implant [143].

Previous researchers have effectively studied 3D-printed implants, such Charcot joints, knee implants, hip implants, spinal cages, radial head prostheses, porous scaffolds in femoral deformity, total talus implants, orthodontic brackets, dental implants, lag screws, heart valves, coronary stents, spine fusion devices, trabecular acetabular cups, and total joint arthroplasty [126,134,137,138]. Figure 8 shows PBF-based 3D-printed biomedical implants.

Figure 8a shows how SLM additive-manufacturing technology was used to fabricate a spinal implant. Researchers must take into account the spinal implant’s biocompatibility, mechanical strength, corrosion resistance, and durability before moving forward with PBF technology. The most typical material used for spinal implants is titanium Ti-6Al-4V. The qualities of titanium include biocompatibility, resistance to corrosion, and good mechanical strength. Spinal implants are also made of stainless steel. Good mechanical strength and corrosion resistance are provided by SS316L [144]. In addition, spinal implants can be made of cobalt chromium [145] in situations where significant mechanical strength is required.

Figure 8b shows PBF technology 3D-printed titanium-based filaments used in biomedical implants, like orthodontic brackets, trauma nails, hip implants, dental implants, coronary stents, spinal cages, coronary stents, lag screws, heart valves, and knee implants [140]. PBF technology is employed to fabricate brackets that are extremely customizable and specifically designed to meet the individual needs of patients. It enhances the therapeutic outcome and decreases the duration [146]. PBF technology can be used to produce complex orthodontic brackets [147]. Titanium is both biocompatible and lightweight, making it well suited for orthodontic purposes [148]. PBF technology has the capability to manufacture titanium trauma nails that possess a high level of functionality. For example, 3D-printed trauma nails, made using PBF technology and coated with bioactive coatings, improve bone regeneration and decrease the likelihood of infection [149,150,151]. PBF 3D-printed hip implants are well suited for the unique anatomy of patients, leading to a natural joint feeling for patients [152], which reduces the need for revision surgery due to implant loosening.

Figure 8c depicts a 3D-printed cellular structure created using SLM for osteoarthritis patients. Recently, numerous researchers have been focusing on cellular structures in an effort to achieve lightweight, high-strength-to-weight-ratio implants [104]. Osteoarthritis is a disabling condition affecting the joints and is marked by the gradual deterioration of cartilage and the bone beneath it. Procedures, such as joint replacement surgery, are invasive and have limitations, especially for younger individuals [153]. To treat osteoarthritis and the associated pain, researchers are looking into customized and minimally invasive methods, including SLM 3D-printed cellular implant plugs [104]. Customized plugs replicate the inherent structure and functionality of healthy joint tissue.

A titanium Ti-6Al-4V acetabular cup manufactured using EBM is depicted in Figure 8d. In a total hip replacement procedure, an acetabular cup is a crucial component. The purpose of the acetabular cup is to replace the weakened or injured native acetabulum [141]. Because each patient has a unique body shape, EBM is used to make a custom acetabular cup that fits perfectly [154]. This improves implant stability, reduces the risk of loosening, and potentially leads to better long-term outcomes [155]. The cup’s porous features facilitate bone ingrowth, securing the implant to the adjacent bone and improving stability [156,157]. Biocompatible Ti-6Al-4V has outstanding osseointegration (bone growth on the implant’s surface) capabilities [158].

Figure 8e shows an EBM 3D-printed femoral appliance made of Co-Cr-Mo. Medical equipment, such as femoral appliances, is utilized in many orthopedic surgeries, mainly for stabilizing or reconstructing the knee joint [159]. The field of orthopedics is undergoing a revolution because of EBM 3D printing, which produces highly functional and customized implants, like Co-Cr-Mo femoral appliances. In the past, femoral appliances were produced using casting or machining methods, which imposed restrictions on customization and intricate shapes [160,161,162]. The utilization of EBM 3D-printing technology to create Co-Cr-Mo femoral appliances signifies a notable breakthrough in the field of orthopedic surgery. EBM 3D-printed Co-Cr-Mo femoral appliances provide services, including customized solutions that enhance the fit, stability, and functionality of implants, resulting in superior patient results and a faster restoration of mobility. The Co-Cr-Mo alloy is suited for femoral applications owing its biocompatibility and high corrosion resistance [163].

Porous pelvic girdles exhibit a porous or osteoporosis-like structure. Osteoporosis is a condition characterized by the loss of bone density and structural deterioration. This condition has been alleviated using 3D-printed cellular implants. Figure 8f shows an EBM-fabricated porous pelvic girdle, specifically a porous right iliac bone made of Ti-6Al-4V [142]. An EBM-manufactured right porous iliac bone can be tailored to match the pelvic anatomy of patients, guaranteeing the optimal fit and stability of the implant. The porous features imitate the natural architecture of the trabecular bone, facilitating bone development and improving the integration of implants [164,165].

The cranial, facial, and maxillofacial regions have been surgically treated and reconstructed using cranio-maxillofacial implants. A cranial–maxillofacial implant was fabricated using SLM–SPS technology by Rahmani et al., as shown in Figure 8g. The integration of SLM and SPS technologies signifies a robust and state-of-the-art method in the realm of cranio-maxillofacial implant fabrication [166]. This novel strategy utilizes the advantages of both techniques to develop customized, highly efficient, and visually appealing implants. The metallic lattice structure and bioactive ceramic powders are used to produce a hybrid metal ceramic [143]. The purpose of these implants is to improve or restore the physical structures of the jaw, face, and skull, frequently in the wake of tumor resections, congenital abnormalities, or other medical disorders.

In addition to these examples, PBF 3D printing can be used to 3D-print spinal spacers, which are used to correct the spine’s height and alignment. Patients with spinal stenosis can move more freely and have less discomfort owing to spinal spacers. PBF technology is utilized to manufacture personalized vertebral bodies to replace destroyed bone in cases of severe spinal injuries [167,168]. PBF 3D printing is utilized for the production of personalized sternal plates, which are employed in the mending of fractured chest bone. Customized plates can be tailored to conform precisely to the curvature of the sternum, hence enhancing the healing process [169,170,171].

Within the fields of tissue engineering and regenerative medicine, scaffold tissue engineering [172] focuses on creating and applying scaffolds to support and promote the formation of new organs or tissues in the human body.

The summary of Section 3.1 is presented in Table 2 below.

Table 2 provides a concise overview of the many types of biomedical implants produced using PBF 3D-printing technology. It outlines the materials that are used, highlights the benefits they offer, and specifies the specific applications they are used for.

### 3.2. Factors Considered for 3D Printing of Biomedical Implants

#### 3.2.1. Implant Design and Material Selection

The important factors to be considered for the 3D printing of biomedical implants are the implant design and material selection. The implant must efficiently reinstate or enhance the impaired function, considering biomechanical aspects, such as the load-bearing capacity and extent of the movement. Materials are commonly selected based on biocompatibility, mechanical strength, and corrosion resistance. Biocompatible materials, such as cobalt–chromium alloys, titanium, and PEEK polymers, are chosen for their robustness, long-lasting properties, and ability to interact harmoniously with human tissues [174]. The different types of materials used in PBF technology and their advantages are explained in Section Materials Used in Biomedical Implants.

#### 3.2.2. Surface Finish

The surface finish is an important factor during the 3D printing of biomedical implants. A good surface finish ensures the overall comfort of patients. In dental implants, the surface roughness is classified into four types based on the average surface roughness. Average surface roughness values below 0.5 µm are considered as smooth; 0.5–1.0 µm, minimally rough; 1.0–2.0 µm, moderately rough; and above 2.0 µm, highly rough [175]. Dental implants currently employ rough titanium surfaces to optimize the bone-to-implant contact (BIC) for enhanced performance [176].

Rajput et al. [177] improved the surface finish of a femoral head fabricated using LPBF by reducing the surface roughness from 14.67 µm to 0.98 µm using H-ECMR and reduced the corrosion rate using the etching process. Following the process of chemical etching, a notable decrease in the corrosion rate was observed, with values dropping from 0.081 mm/year to 0.0103 mm/year. This technique resulted in an enhancement in the biocompatibility of the femoral head.

Sandblasting was used by Ziółkowska et al. [178] to lower the average surface roughness of Ti6Al4V implants manufactured via EBM from 31.77 ± 0.94 µm to 24.27 ± 1.36 µm.

#### 3.2.3. Accuracy and Precision

When it comes to 3D printing biomedical implants, accuracy and precision are crucial factors. These two crucial factors determine the implants’ performance, long-term stability, and patient safety [179]. Designed models and 3D-printed models need to be similar. Inaccuracies can lead to incompatibility with the patient’s anatomical structure, resulting in instability, pain, and the possibility for requiring revision surgery [180,181]. PBF technologies can provide 3D-printed implants that are incredibly precise and accurate [182]. However, to achieve accuracy and precision, it is crucial to calibrate the PBF apparatus. Furthermore, other approaches, such as employing high-resolution scanning of the human anatomy, utilizing modern 3D-printing techniques, implementing rigorous quality control and inspection protocols, and engaging in ongoing research and development, are employed to achieve optimal levels of accuracy and precision [183,184,185]. A well-made 3D-printed component fits perfectly in a specific implant location.

#### 3.2.4. Sterility and Cleanliness

Sterilization is a crucial stage in the process of 3D-printing biomedical implants. Implants must be free from all microbial contamination to prevent post-surgical infection [186]. To avoid contamination, the environment in which implants are made must be sterile. To comply with regulatory criteria and provide safe implants, proper cleaning and sterilization methods are necessary. The challenges in the sterilization of 3D-printed implants include the sterilization of complex structures. In contrast to conventional implants that have uncomplicated forms, 3D-printed implants might possess intricate internal structures and porous designs, posing challenges for sterilizing agents to properly access all the surfaces [187,188]. Common sterilization methods for 3D printing include gamma irradiation [189], steam sterilization [190], hydrogen peroxide gas plasma [191], and ethylene oxide [192].

#### 3.2.5. Testing and Quality Control

Crucial aspects to take into account for the 3D printing of biomedical implants are quality control and testing. The structural integrity and functionality of 3D-printed implants are guaranteed by quality control and testing. This may encompass several procedures, such as the mechanical testing, imaging, and inspection of the finished product. Tensile testing, three-point bending, and hardness testing are the mechanical-testing methods that are the most frequently utilized. Scanning electron microscopy (SEM) is employed to assess the microstructural characteristics of 3D-printed components [193]. The results of cytotoxicity tests indicate whether implants will be harmful to humans [194,195].

The summary of Section 3.2 is presented in Table 3 below.

The summary in Table 3 led to the conclusion that the implant’s design, surface finish, accuracy, sterility, and testing are the five main elements that must be carefully considered when 3D-printing biomedical implants. By maintaining a balance between these variables, implants are guaranteed to be biocompatible, functional, and anatomically correct for the patient, reducing the risk of surgery and increasing long-term success.

## 4. Advantages of PBF 3D Printing in Precision Manufacturing

### 4.1. High Degree of Precision

PBF 3D-printing technology can be used to create parts that are incredibly accurate and have tight tolerances. Because of their extreme precision, PBF 3D-printed parts fit together, and the printed item operates smoothly. PBF 3D printing has been used to produce intricate medical implants that precisely fit the anatomy of the patient [196,197]. The layer’s typical thickness is between 20 and 200 µm [55,66,72,83], which permits the creation of complex and incredibly detailed components. In fields where extreme precision is required, like aerospace and medical devices, this degree of precision (20–100 µm) is useful. Precision manufacturing encompasses a wide range of applications, from crafting delicate watch gears to fabricating sophisticated aerospace components, owing to its high level of accuracy [198,199]. PBF enables the construction of very intricate and reliable components with assurance.

### 4.2. Complex Geometries

PBF technology allows researchers and users to manufacture complex geometries and shapes required in unique areas where conventional manufacturing is impossible. This capability helps researchers to easily produce complex internal structures, lattices, and honeycomb patterns [200,201,202,203,204].

### 4.3. Reduced Waste

PBF technology employs an additive-manufacturing approach, wherein materials are incrementally deposited layer by layer to fabricate a desired component, as opposed to the conventional subtractive-manufacturing method, which involves material removal [25]. The amount of waste material generated by PBF technology is less than that generated by other subtractive-manufacturing technologies [205]. This aspect holds particular significance within the realm of precision manufacturing, given the considerable impact that material costs can have.

### 4.4. Quick Iteration

PBF technology makes it possible to prototype and iterate quickly. Without the need for expensive molds or equipment, iterations can be completed rapidly. This flexibility is crucial for sectors that need quick development [206]. PBF 3D printing eliminates the need for time-consuming machine setups and complex tooling [207]. The 3D models can be readily refined through the utilization of modeling software. PBF facilitates the testing of novel concepts and enables efficient iterations.

### 4.5. Short Lead Times

Compared with conventional manufacturing methods, PBF is an innovative technology that produces accurate components with reduced production times and has revolutionized industry [208]. The most important aspect of PBF is that it does not require any setup, tools, or machining, all of which take a lot of time in traditional manufacturing processes [196]. The manufacturing process of precision parts in conventional manufacturing entails multiple phases. The setup phase commences with the preparation of the machinery for the production process. Following this, tooling occurs, which entails the creation, configuration, and maintenance of the instruments utilized in the production process. Machining, the last stage, is where the physical fabrication of the components occurs. Extended lead periods are the result of the substantial time and resources required for each of these stages.

In contrast, PBF technology eliminates these stages, thereby streamlining the procedure. PBF fuses sections of a powder bed into a solid part using a high-energy source, such as an electron beam or laser, as opposed to undergoing preparation, tooling, and machining [209]. Layer by layer, this procedure is repeated until the complete component is manufactured. Compared with conventional manufacturing techniques, PBF is consequently capable for manufacturing intricate and accurate components in a minuscule amount of time [210].

### 4.6. Reduced Assembly

PBF technology represents a significant manufacturing advancement when it comes to the ability to produce an entire assembly in a single print. This process obviates the necessity for joining multiple components, a procedure frequently employed in conventional manufacturing [211,212]. Conventional approaches frequently entail the fabrication of discrete components, which are subsequently merged to constitute an end product. This not only necessitates further investment of time and resources but also presents the possibility of errors and weaknesses.

In conventional assembly, each component must be manufactured with extreme precision before being assembled. Inaccuracies may arise during this joining procedure as a result of tolerance stacking, alignment issues, or human error [213]. Furthermore, the interfaces where constituent parts are joined frequently transform into areas of weakness in the end product, which may compromise the integrity of the structure.

On the contrary, PBF technology enables the simultaneous printing of an entire assembly. As a result, there is no requirement to connect multiple components; the end result is a solitary, solid piece [214,215]. Consequently, the likelihood of errors and risks arising from the assembly procedure is substantially diminished. This results in products that possess enhanced precision, durability, and dependability.

Furthermore, substantial time and financial savings are realized when an entire assembly is printed in a single pass [216]. It obviates the necessity for supplementary procedures, such as component-specific assembly and quality-control inspections. As a result, PBF technology is an exceptionally effective and economical manufacturing solution.

### 4.7. No Tooling Cost Required

PBF technology is a revolutionary advancement in the manufacturing sector, specifically in the fabrication of intricate components. Conventional manufacturing techniques frequently depend on costly dies, tools, and molds to shape and fabricate components [217]. These instruments are not only expensive to create and maintain but also restrict the level of intricacy achievable in manufactured parts.

But this paradigm has undergone a significant shift because of PBF technology. In contrast to traditional techniques, PBF eliminates the need for dies, tools, and molds. Instead, it fuses portions of a powder bed together to form a solid object using a high-energy source, like a laser or electron beam. Layer by layer, this process is continued until the part is completed. Because of this, PBF is able to create intricately geometrized, complicated parts that would be difficult, if not impossible, to fabricate with conventional production techniques.

The elimination of the requirement for costly tools and molds results in a substantial decrease in manufacturing expenses [206]. Not only are the initial expenses for creating and upkeeping these tools minimized but also the expenses related to their storage and eventual replacement [218]. Moreover, the capability to manufacture components as needed eliminates the necessity for maintaining a substantial stock of parts, thus further decreasing expenses.

Furthermore, PBF technology provides advantages in terms of cost effectiveness, as well as enhanced efficiency and flexibility. The capacity to generate components as needed enables a closer synchronization between production and consumption, resulting in waste reduction and enhanced efficiency. Furthermore, the adaptability of PBF technology allows for modifications to be included in the design of components without requiring expensive and time-consuming alterations to tools and molds [219].

### 4.8. Inventory Reduction

PBF technology is a revolutionary advancement in the manufacturing industry, specifically owing to its capacity to create parts as needed, resulting in a substantial decrease in inventory [220]. Conventional manufacturing methods typically need the maintenance of a substantial stock of components, along with specialized machinery, molds, or dies. These objects not only occupy a significant amount of physical space but also immobilize financial resources that could be allocated to other areas of the organization.

Nevertheless, PBF technology disrupts this paradigm by facilitating the fabrication of components precisely at the moment they are required. The ability to produce items as needed eliminates the necessity for storing a substantial quantity of parts in preparation for future requirements [221]. Alternatively, components can be manufactured with precision when needed, resulting in a more optimal utilization of resources and a substantial decrease in the need for storage space.

The summary of Section 4 is presented in Table 4 below.

From Table 4, it was concluded that precision manufacturing is being revolutionized by PBF 3D printing, which produces intricate, highly precise parts with less waste and shorter lead times. Powerful for creative and effective production, PBF 3D printing reduces inventory, eases assembly, does away with expensive tooling, and allows for quick design iterations.

## 5. Challenges and Limitations

### 5.1. Technical Challenges

**Figure 9:**
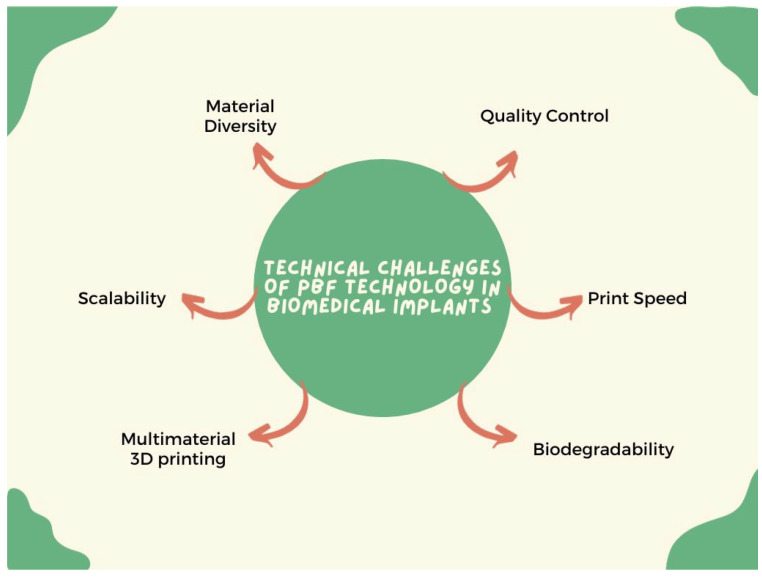
Illustration of the technical challenges of PBF technology in fabricating biomedical implants.

Figure 9 depicts the technical difficulties associated with PBF technology in fabricating biomedical implants.

#### 5.1.1. Material Diversity

PBF technology utilizes a restricted diversity range of materials. The materials used in biomedical applications must be biocompatible with the human body and natural tissues. A major concern in the 3D printing of biological implants is the choice of the binder. The binder ought to be easily available, biodegradable, nontoxic, and easy to handle [222]. The materials that are utilized have a substantial impact on the quality of biomedical implants [223]. There are binders, like polymeric binders, that can be found, but their removal may involve additional heating or chemical postprocessing, which could compromise their biocompatibility [212,224,225,226,227]. Although wax-based binders are easy to print on and remove, there are concerns over the potential for wax residues to be present and their potential long-term compatibility with living organisms [212,225].

#### 5.1.2. Biodegradability

The management of biodegradation kinetics is of the utmost importance for magnesium- and iron-based materials because premature degradation can result in an untimely decline in mechanical strength, impeding the complete recovery of tissues or functions [228]. The utilization of degradable biomedical implants featuring bioactive ceramic coatings has the potential to expedite the process of wound healing [229]. The biodegradability of the materials used in biomedical implants must match the body’s healing process to promote the maximum healing and minimize side effects [230].

#### 5.1.3. Multi-Material 3D Printing

**Figure 10:**
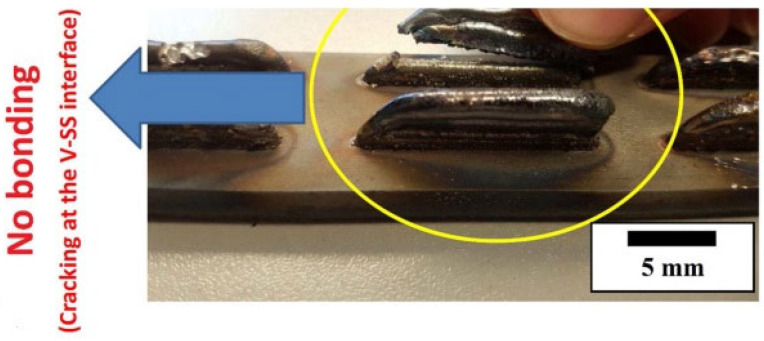
Illustration of the inadequate bonding that occurred as a result of depositing zirconium (Zr) on an A410-L stainless-steel substrate using titanium and vanadium as interlayers (shown by the yellow circle), adapted from [233].

Using multiple materials in a single print is difficult with PBF technology [182], especially for organ printing. Printing many materials in 3D organ printing poses challenges because of the different properties of the materials. Precision control over materials is necessary for organ printing [231]. Material thermal properties vary, and the combination of multiple materials can result in cracking, porosity, and poor interlayer adhesion [232]. The recovery of leftover materials and material recycling are additional difficulties in multi-material PBF 3D printing [35]. In Figure 10, the yellow circle illustrates the inadequate bonding that occurred as a result of depositing zirconium (Zr) on an A410-L stainless-steel substrate using titanium and vanadium as interlayers. Thermodynamic instability leads to improper bonding, which increases the likelihood of cracking and weakens the adhesion between the layers. The different crystal structures of stainless steel and zirconium may contribute to stress accumulation and eventually lead to poor bonding [233].

A variety of factors contributed to the formation of the crack, including the intermetallic formation, the difference between the thermal expansion coefficients of the two metals, the susceptibility to environmental cracking, the atmospheric quality, the rate of cooling, the ductility of the deposited metallic material, the variation in the melting points, the thermal conductivity of the substrate, the thickness and temperature of the substrate, the laser feed rate, and the laser power density [233].

#### 5.1.4. Printing Speed

Complex parts take more time to print [234]. Although increasing the printing speed is desirable, it may come at the expense of the printing quality. PBF technology scans faster, resulting in the appearance of larger, irregularly shaped pores [235].

After printing each individual layer in PBF, the powder must be dispersed across the print bed. This powder takes time to disperse throughout the print bed and influences the printing speed.

#### 5.1.5. Scalability

The mass production of parts using PBF technology is impossible owing to the printing time and cost [236]. The printing volume of the PBF 3D printer is quite low, which reduces the mass-production efficiency [237]. Postprocessing is sometimes necessary after 3D printing, which adds time and cost to the process. Presently, PBF technology is encountering obstacles in large-scale manufacturing. However, ongoing research endeavors hold the potential to enhance the efficiency and cost effectiveness of PBF in the future [238].

#### 5.1.6. Quality Control

The implementation of inspection procedures is crucial in guaranteeing the quality of 3D-printed implants. PBF 3D printing presents challenges in terms of controlling material qualities and process parameters. Any variance in these results in defects [239].

In PBF technology, the final mechanical strength of products is adversely affected by the absence of density and porosity. The loss of dimensional correctness and structural integrity is the result of the quick heating and cooling processes. The implementation of quality controls for the process parameters of PBF has been enhanced by the utilization of artificial intelligence and machine-learning techniques, resulting in improved part quality [240,241].

### 5.2. Regulatory and Safety Concerns

PBF technology requires careful consideration of safety and regulatory issues to guarantee patient safety, product efficacy, and regulatory compliance. For PBF 3D-printed biomedical implants, obtaining regulatory approval is the main concern. Medical devices, including implants, are regulated by the FDA in the United States [138]. To receive FDA approval for their products, PBF technology manufacturers have to successfully complete the regulatory procedure.

**Figure 11:**
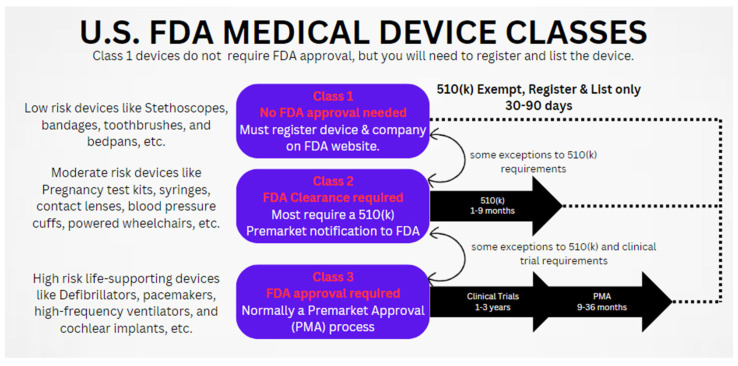
FDA medical-device classes, adapted and modified from [242].

As shown in Figure 11, the FDA classifies medical devices into three classes [242]. Low-risk items, such as bandages, tongue depressors, pads, and toothbrushes, are included in Class 1. Class 1 devices have an easy-to-use design and pose no danger to patients. Medical device registration and listing are mandatory; however, FDA approval is not needed for Class 1 devices [138].

Devices classified as Class 2 carry considerable risk. More regulatory control is needed for Class 2 devices than for Class 1 devices. Class 2 includes devices with a moderate level of danger, such as surgical gloves, CPR equipment, and ultrasounds. Class 2 devices are more complicated to develop and may be more dangerous if misused or not utilized correctly. FDA clearance is required. Class 2 devices often require pre-market notification (510(k)) to demonstrate substantial equivalence to a legally marketed device [243,244,245].

High-risk life-supporting devices come under Class 3 devices. The strictest regulatory controls are necessary for Class 3 devices. Class 3 devices are complex in design and used for life-supporting purposes and have a higher potential for harm if they fail [246]. Class 3 devices include objects like pacemakers, implants, and implanted defibrillators. Pre-market approval (PMA), which is obtained from the FDA after extensive clinical trials proving safety and efficacy, is necessary for Class 3 devices [243,244,245].

Regarding safety, substances must be biocompatible with the human body. The human body reacts negatively to certain material properties. Therefore, considerable attention should be given when choosing materials [226]. Second, to eliminate any microbiological contamination, 3D-printed items must be sterilized. One method that is frequently used to eliminate microorganisms from the surfaces of 3D-printed objects is steam sterilization, which uses saturated steam at high pressures [247].

## 6. Future Prospects and Conclusion

From this study, it is evident that PBF technology holds significant potential for the future of biomedical implants. Despite being in its early stages, the use of multiple materials in PBF is a promising area for future research and development. However, the high cost of PBF technology is a current drawback that needs to be addressed.

Increasingly, surgeons are utilizing patient-specific surgical guides and instruments created through PBF, leading to more precise treatments and fewer procedures. Future advancements in this technology could further enhance surgical planning and performance.

In addition, PBF technology could revolutionize the pharmaceutical sector by enabling the creation of tailored medication formulations and dosage forms. This could significantly advance personalized medicine and improve the efficacy of drug delivery systems.

One exciting prospect is the potential for manufacturing biodegradable implants using PBF. These implants, designed to gradually dissolve within the body, could release drugs or promote tissue regeneration over time, eliminating the need for removal surgery.

In conclusion, PBF technology has substantial potential not only in biomedical applications but also in industrial sectors, such as automotive and aerospace. Looking ahead, we can anticipate advancements in PBF technology that include reduced dimensions, lower costs, and enhanced multi-material capabilities.

## Figures and Tables

**Table 1:** Biomedical materials and their advantages.

Material	Advantages
Titanium and its alloys	High degree of biocompatibility and minimal allergic response risk [118]
	High strength-to-weight ratio and structural integrity [119]
	Corrosion resistance and ensures the longevity of the implant in the body [120]
	Good osseointegration encourages the formation and attachment of new bone [121]
Co-Cr-based alloys	Useful for articulating surfaces owing to high wear resistance [122]
	Excellent biocompatibility and corrosion resistance [123,124]
	A good fit for load-bearing implants, including hip and knee replacements [125]
NiTi alloy	Dynamic implants with unique shape memory and superelasticity [126]
	Biocompatible and corrosion resistant [126]
	Ideal for vascular implants, devices for orthodontics, and stents [126]
Stainless steel	Biocompatibility and high resistance to corrosion [127]
	The availability of several grades designed to meet implant requirements [128]
	Economical choice [129]
Magnesium and its alloys	Lightweight and density is similar to that of bone [130]
	Biodegradable; slowly metabolized by the body over time [131]
	Appropriate for utilization in temporary implantation scenarios, such as bone fixation devices [132,133]
AlSi10Mg	The material exhibits favorable mechanical properties by effectively integrating both high strength and low density [134]
	Corrosion resistant [135]
	High specific strength [136]

**Table 2:** Summary of PBF 3D-printed biomedical implants.

Implant	Material	Advantages	Applications	References
Spinal	Ti-6Al-4V; SS316L; Co-Cr	Biocompatible; mechanically strong; corrosion resistant	Customizable; improved fit and stability	[126,134,137]
Orthodontic brackets	Titanium	Highly customizable; reduced treatment time	Improved therapeutic outcomes	[146,147]
Trauma nails	Titanium (coated with bioactive coating)	High degree of functionality; promote bone regeneration; reduce infection	Osteoporosis treatment	[149,150,151]
Hip	Titanium	Natural joint feeling; reduced probability for requiring revision surgery	Improves patient experience and long-term outcomes	[152]
Cellular implant plugs for osteoarthritis	Titanium	Lightweight; replicate healthy joint tissues	Minimally invasive treatment; promote cartilage regeneration	[104]
Acetabular cup	Ti-6Al-4V	Customized fit; improved stability; bone ingrowth	Total hip replacement	[141,154,155,156,157]
Femoral appliance	Co-Cr-Mo	Customized; enhanced fit and functionality	Faster restoration of mobility after knee surgeries	[159,160,161,162,163]
Porous pelvic girdle	Ti-6Al-4V	Customized fit; stability; facilitates bone growth	Treatment for osteoporosis	[142,164,165]
Cranio-maxillofacial implant	Hybrid metal ceramic	Customized; aesthetically pleasing; improved osseointegration	Jaw, face, and skull reconstruction	[143,166]
Spinal spacers	Titanium	Correct spinal height and alignment	Reduced pain and improved mobility in spinal stenosis patients	[173]
Vertebral bodies	Titanium	Customizable; replace destroyed bone in spinal injuries	Spinal reconstruction	[167,168]
Sternal plates	Titanium	Customized, enhance healing process	Repair fractured chest bone	[169,170,171]

**Table 3:** Summary of factors considered for the 3D printing of biomedical implants.

Factor	Description	Importance	Examples	References
Implant design and material selection	Matching function and biocompatibility	Crucial for long-term success and patient safety	Co-Cr alloy; titanium; PEEK polymers	[174]
Surface finish	Patient comfort and osseointegration	Affects bone–implant contact and infection risk	Rougher for dental implants; smoother for joint implants	[175,176]
Accuracy and precision	Proper fit and function	Avoids revision surgery and complications	High-resolution scanning; modern printing; quality control	[179,180,181,182,183,184,185]
Sterility and cleanliness	Prevents post-surgical infection	Essential for patient safety	Gamma irradiation; steam; hydrogen peroxide; ethylene oxide	[186,187,188,189,190,191,192]
Testing and quality control	Structural integrity and functionality	Guarantees safety and performance	Mechanical testing; imaging; cytotoxicity testing	[193,194,195]

**Table 4:** Summary of advantages of PBF 3D printing in precision manufacturing.

Advantage	Description	Importance	Examples	References
High degree of precision	Tight tolerances and accurate parts	Minimizes assembly problems and improves functionality	Medical implants; aerospace components	[196,197]
Complex geometries	Create intricate internal structures and unique shapes	Impossible with traditional manufacturing processes	Lattices; honeycomb patterns	[200,201,202,203,204]
Reduced waste	Additive approach uses only needed material	Minimizes material costs and environmental impact	compared to subtractive manufacturing	[205]
Quick iteration	Rapid prototyping and design changes	Saves time and resources during development	No need for expensive tooling or setup	[206,207]
Short lead times	Faster production compared to traditional methods	Reduce time to market and improve production efficiency	Eliminate setup, tooling, and machining stages	[196,208]
Reduced assembly	Prints entire assemblies in one piece	Minimizes errors and weaknesses; improves product quality	Avoids joining multiple components and associated risks	[211,212]
No tooling cost required	Eliminates expensive dies, tools, and molds	Reduces manufacturing costs and increases flexibility	Creates complex parts that are impossible to create with traditional methods	[217,218]
Inventory reduction	Creates parts on demand, minimizing stock	Frees up space and resources; improves efficiency	Eliminates need for storing large quantities of parts	[220,221]

## Data Availability

Not applicable.

## Abbreviations

3D	three dimensional
AM	additive manufacturing
AlSi10Mg	aluminum–silicon–magnesium alloy
BIC	bone-to-implant contact
Co-Cr-based alloy	cobalt–chromium-based alloy
Co-Cr-Mo	cobalt–chromium–molybdenum
CPR	cardiopulmonary resuscitation
DMLS	direct metal laser sintering
EBM	electron beam melting
FDA	Food and Drug Administration
H-ECMR	hybrid electrochemical-assisted magnetorheological
HP	Hewlett-Packard
LPBF	laser powder bed fusion
MJF	multi-jet fusion
NiTi	nitinol
PBF	powder bed fusion
PMA	pre-market approval
SEM	scanning electron microscopy
SHS	selective heat sintering
SLM	selective laser melting
SLM–SPS	selective laser melting–spark plasma sintering
SLS	selective laser sintering
SS316L	stainless steel 316L
Ti-6Al-4V	titanium–6% aluminum–4% vanadium alloy
